# Protective Role of the ACE2/Ang-(1–9) Axis in Cardiovascular Remodeling

**DOI:** 10.1155/2012/594361

**Published:** 2012-01-19

**Authors:** María Paz Ocaranza, Jorge E. Jalil

**Affiliations:** Cardiovascular Diseases Division, School of Medicine, Faculty of Medicine, Pontificia Universidad Catolica de Chile, Chile

## Abstract

Despite reduction in cardiovascular (CV) events and end-organ damage with the current pharmacologic strategies, CV disease remains the primary cause of death in the world. Pharmacological therapies based on the renin angiotensin system (RAS) blockade are used extensively for the treatment of hypertension, heart failure, and CV remodeling but in spite of their success the prevalence of end-organ damage and residual risk remain still high. Novel approaches must be discovered for a more effective treatment of residual CV remodeling and risk. The ACE2/Ang-(1–9) axis is a new and important target to counterbalance the vasoconstrictive/proliferative RAS axis. Ang-(1–9) is hydrolyzed slower than Ang-(1–7) and is able to bind the Ang II type 2 receptor. We review here the current experimental evidence suggesting that activation of the ACE2/Ang-(1–9) axis protects the heart and vessels (and possibly the kidney) from adverse cardiovascular remodeling in hypertension as well as in heart failure.

## 1. Introduction

All epidemiological studies show that the risk of adverse cardiovascular (CV) outcomes, such as stroke, myocardial infarction (MI), heart failure (HF), and kidney disease [1], increase progressively with increasing blood pressure (BP). On the other hand, clinical trials demonstrate that lowering BP reduces such risks [1]. All antihypertensive medications lower BP, but specific drug classes display effects beyond BP reduction (pleiotropic effects) that might contribute to cardiovascular risk reduction. 

Remodeling of the cardiovascular structure occurs in response, not only to changes in BP and flow, but also to modifications in the neurohormonal environment, in which the rennin-angiotensin-aldosterone system (RAAS) exerts a most predominant influence [2].

The RAAS is a major regulator of BP [3, 4]. In addition, the RAAS has a role in the vascular response to injury and inflammation [4]. Chronic RAAS activation, through both angiotensin (Ang) II and aldosterone, leads to hypertension and perpetuates a cascade of proinflammatory, prothrombotic, and atherogenic effects associated with end-organ damage [3, 4]. Based on these facts, several drugs have been developed that work by (a) reduction of Ang II levels, (b) inhibition of the Ang II type 1 receptor (AT1R), (c) blockade of the aldosterone receptor, and (d) renin receptor blockade [5, 6]. During the last 25 years several clinical trials have shown the benefits with these drugs that inhibit the RAAS with regard to BP reduction, regression of cardiac hypertrophy, prevention of kidney damage and reduction of cardiovascular morbidity reduction in hypertensive patients. Besides, with most of these RAAS blockers, quality of life as well as survival has been significantly improved in patients with heart failure. Consequently, the RAAS is currently a main therapeutic target in hypertension treatment [3, 4]. Aggressive BP control improves outcomes in patients with CV disease, stroke, and nephropathy and might have beneficial effects beyond BP lowering [7]. 

 Despite the reduction of CV events and end-organ damage with the current pharmacologic strategies, CV disease remains the primary cause of death in the world, and more than 94,000 Americans annually experience progression to end-stage renal disease (ESRD). As population ages, the proportion affected by end-organ damage is expected to grow [8]. Thus, it is most relevant to find new molecules in order to prevent and reduce hypertension as well as pathologic CV and kidney remodeling and dysfunction. In this regard, activation of the new ACE2/Ang-(1–9) pathway seems to counterbalance the damage due to the RAAS system activation. 

 We review here the current experimental evidence suggesting that activation of the ACE2/Ang-(1–9) pathway protects the heart and vessels (and possibly the kidney) from adverse cardiovascular remodeling in hypertension as well as in heart failure. 

## 2. Angiotensin-Converting Enzyme 2

The discovery of angiotensin-converting enzyme homologue, ACE2, added further complexity to the main axis of the RAAS, in which Ang II and its forming enzyme ACE play major roles [9, 10]. A growing body of evidence points to a possible promising role for this new member of the RAAS by opposing to the effects of the main axis [11, 12]. ACE2 has dramatically changed the direction of cardiovascular and renal research in view of the pivotal role of this enzyme in the regulation of the RAAS [12, 13]. 

ACE2 is the newest member of the RAAS and shares approximately 40% similarity with the somatic form of ACE [9, 10]. ACE2 is a membrane-bound carboxypeptidase and its cellular and tissue distribution is different from that of ACE. While ACE is expressed in the endothelium throughout the vasculature, ACE2 is distributed in tissues with the most abundant expression in heart, kidney, lung, small intestine, and testis [14]. ACE2 can be released into the circulation and urine by shedding [15]. Tumor necrosis factor-alpha-converting enzyme (TACE/ADAM17) is the sheddase responsible for the ectodomain cleavage and shedding of ACE2 [16]. 

However, normal ACE2 enzymatic activity in plasma is very low, probably due to the presence of an endogenous inhibitor [17–19]. ACE2 is different from ACE in both substrate specificity and functions [9, 20, 21]. ACE2 can form (a) Ang-(1–7) through hydrolysis of Ang II and (b) Ang-(1–9) through hydrolysis of Ang I. This last reaction is negligibly slow and is several hundred times slower than Ang II hydrolysis by ACE2 to form Ang(1–7)—a vasodepressor peptide counterbalancing the vasopressor effect of Ang II [20, 21]. Ang-(1–7) can be subsequently converted to Ang-(1–5) by ACE [9, 20] or by neutral endopeptidases [9], while Ang-(1–9) may be converted to Ang-(1–7) by ACE [9]. There is little evidence proving the existence of alternative hydrolysis of Ang-(1–9) to Ang II in some tissues. Drummer et al. [22] proved that homogenates of rat kidney, and in a lesser extent of lung, convert Ang-(1–9) to Ang II due to an ACE-independent aminopeptidase and N-like carboxypeptidase. Singh et al. [23] confirmed that the pathway Ang I-Ang-(1–9)-Ang II really exists in glomeruli of streptozotocin-induced diabetes mellitus rats. Moreover, in human heart tissue the main products of Ang I degradation are both Ang-(1–9) and Ang II generated by heart chymase, ACE and a poorly identified carboxypeptidase A [24]. Although the data proving the existence of alternative pathways of Ang II production, in clinical practice we can still block only ACE or AT1R. 

ACE2 does not act on bradykinin metabolism and its activity is not inhibited by classic ACE inhibitors (ACEIs) [9]. Thus it has been proposed that ACE2 activity may counterbalance the effects of ACE by preventing the accumulation of Ang II in tissues where both ACE2 and ACE are expressed [25, 26]. ACE2 has several biological substrates and it is considered a multifunctional enzyme. Acting as a monocarboxypeptidase, it cleaves several other non-RAAS peptides which have roles in maintaining cardiovascular homeostasis such as (des-Arg9)-bradykinin, a member of the kininogen-kinin system [13]. (des-Arg9)-Bradykinin is formed from bradkinin by the action of carboxypeptidases and is an agonist of the B1 receptor, which is induced after tissue injury [27]. Bradykinin, a vasodilator which acts through the B2 receptor, is produced from its precursor kininogen by kallikrein and is degraded by ACE [13]. While, degradation of bradykinin by ACE is known to be an important aspect of BP regulation, the significance of the degradation of (des-Arg9)-bradykinin by ACE2 remains to be established. In addition to (des-Arg9)-bradykinin, ACE2 is also able to degrade apelin-13, a peptide proposed to cause vasoconstriction and known to regulate fluid homeostasis, and other non-RAAS peptides such as kinetensin, dynorphin A and neurotensin [20].

For a long time, Ang-(1–7) was thought to be devoid of biological activity, in spite of early reports on biological effects [28]. The importance of Ang-(1–7) was emphasized by the discovery of ACE2. Ang-(1–7) has been shown to release vasopressin as effectively as Ang II from neurohypophyseal explants [28] and to have actions opposing those of Ang II, namely vasodilation, antitrophic effects and implications of vasodilation caused by bradykinin [29, 30]. 

Several experiments suggest an important interaction between Ang-(1–7) and prostaglandin-bradykinin-nitric oxide (NO) systems. Ang-(1–7) binds to the Mas receptor (G protein-coupled receptor) which mediates vasodilating and antiproliferative actions of this peptide [31]. The Mas receptor can hetero-oligomerize with the AT1 receptor and acts as a physiological antagonist of Ang II [32]. Studies revealed that Ang-(1–7) activated endothelial nitric oxide synthase and NO production via Akt-dependent pathways [33]. Furthermore, Tallant et al. [34] showed that the presence of an antisense probe directed against Mas abolished the Ang-(1–7)-induced inhibition of protein synthesis in cardiomyocytes. This study also revealed that Ang-(1–7) decreased serum-stimulated ERK1/ERK2 mitogen-activated protein kinase activity, a response that was blocked by D-Ala 7-Ang-(1–7), an antagonist of Mas receptor. 

Ang II binds with high affinity to two different receptor subtypes—AT1R and AT2R—which are members of the seven-transmembrane-domain G-protein-coupled receptors (GPCR) superfamily, through Gq and Gi, respectively [35]. Whereas the AT1R mediates most of the recognized actions of Ang II, it appears that the AT2R opposes, in part, to the effects mediated by the AT1R. As the AT2R is expressed in adult tissues in smaller amounts than the AT1R, the actions and cell signaling of AT2R have been less well characterized than those of AT1R [36–38]. Current knowledge suggests that AT2R stimulation mediates vasodilation, antigrowth, proapoptotic and antiinflammatory effects [39, 40]. Hence, the AT2R can modulate cardiovascular remodeling as well as progression of atherosclerosis. 

 AT2R stimulation activates the NO-cGMP-dependent pathway [41]. This occurs either directly or indirectly through bradykinin or by increased endothelial NOS activity or expression. AT2R activation is associated with phosphorylation of JNK, PTPs, I*κ*B*α* (inhibitor of NF-*κ*B), and the transcription factor ATF2, and dephosphorylation of p38MAPK, ERK1/2, and STAT3, which are linked to antiproliferative and antiinflammatory effects and apoptosis [38, 42–44]. AT2R may induce relaxation by opening large-conductance Ca^2+^-activated K^+^ channels (BKCa) [45] and by negative regulation of the vascular Rho A/Rho kinase pathway. The AT2R also enhances the activity of tyrosine phosphatases and vanadate-sensitive phosphatases MKP1 (DUSP1), SHP1 (PTPN6) and PP2A [46, 47]. 

## 3. Ang-(1–9)

There is little information in the literature with respect to Ang-(1–9) probably because this peptide was initially thought to be active only after conversion to Ang-(1–7). Ang-(1–9) can be generated by several carboxypeptidase-type enzymes including ACE2 or cathepsin A [48, 49]. Ang-(1–9) is present in healthy volunteers, in patients or in animals treated with ACE inhibitors (ACEIs) or AT1 receptor blockers (ARBs) [50–52], and its circulating levels are increased by pathological conditions (i.e., early after MI) [51]. However, very little is currently known about Ang-(1–9) biological effects [50, 53]. 

Initial studies showed that incubation of Chinese hamster ovary cells (CHO) with Ang-(1–9) potentiated the release of arachidonic acid by [Hyp^3^Tyr(Me)^8^]BK, elevated [Ca^2^]i and also resensitized the B2 receptor desensitized by BK [48]. At the same time, Jackman et al. [54] showed in CHO cells and in human pulmonary endothelial cells that Ang-(1–9) was significantly more active than Ang-(1–7) enhancing the effect of an ACE-resistant bradykinin analogue on the B2 receptor and that Ang-(1–9) also augmented arachidonic acid and NO release by kinin [54].

Some studies have suggested that Ang-(1–9) may be an endogenous inhibitor of ACE. Donoghue et al. [9] proposed that Ang-(1–9) is a competitive inhibitor of ACE because it is by itselfan ACE substrate. Under conditions of ACE inhibition, such as after long-term administration of an ACEI in rats, Ang-(1–9) levels increased in plasma and kidney [50, 53]. This increase in Ang-(1–9) steady-state levels could be due to decreased catabolism of Ang-(1–9) by ACE. Conversely, the increased levels of Ang-(1–9) could be due to increased production by ACE2 as a result of increased availability of Ang I substrate. These results indicate that an alternate pathway of Ang I metabolism by ACE2 exists and that this pathway may be amplified in the presence of ACE inhibitors. 

To determine whether Ang-(1–9) is active per se or it becomes active only after conversion to Ang-(1–7), Chen et al. [55] examined the metabolism of Ang I, Ang-(1–9) and Ang-(1–7) in stably transfected CHO cells that express human ACE and human bradykinin B2 receptors coupled to green fluorescent protein (B2GFP). They found that Ang-(1–9) was hydrolyzed 18 times slower than Ang I and 30% slower than Ang-(1–7). Ang-(1–9) inhibited ACE and it resensitized the desensitized B2GFP receptors, independently of ACE inhibition [55]. This is reflected by release of arachidonic acid through a mechanism involving cross-talk between ACE and B2 receptors. They concluded that Ang-(1–9) enhanced bradykinin activity, probably by acting as an endogenous allosteric modifier of the ACE and B2 receptor complex. Therefore, when ACE inhibitors block conversion of Ang I, other enzymes like ACE2 can still release Ang I metabolites like Ang-(1–9) and enhance the efficacy of ACEIs. 

 Recently, Flores-Muñoz et al. [56] using radioligand binding assays observed that Ang-(1–9) is able to bind the Ang II type 2 receptor (AT2R) (pKi = 6.28 ± 0.1). They demonstrated that Ang-(1–9) and not Ang II, affected hypertrophy through the AT2R, as PD123319 (an AT2 receptor blocker) did not alter Ang II-mediated growth but did block the effects of Ang-(1–9). Despite having ~100-fold lower affinity than Ang II for the AT2R [57], the selective AT2R activity of Ang-(1–9) is not inconsistent with current pharmacological models of G protein-coupled receptor signalling and activation. Indeed, the concept of functional selectivity, where individual receptor ligands have the capacity to selectively stabilize conformations which lead to distinct signalling outcomes [57–59], is supported by a previous study in which the critical amino acids and the mode of binding of ligands at the AT1R and AT2R were investigated [60]. While agonist activation of the AT1R was particularly sensitive to peptide modifications that disrupted contact points between Ang II and its receptor, substitutions within Ang II were far better tolerated by the AT2R [60]. The AT2R exists in a relaxed conformation and Ang II therefore binds to multiple indistinct contact points [60]. Since Ang-(1–9) contains the entire Ang II sequence plus a C-terminal histidine, these observations indicate that this difference may stabilize the AT2R in a conformation able to counteract hypertrophic signalling in cardiomyocytes. Flores-Muñoz et al. [56] did not observe functional competition between Ang II and Ang-(1–9) at the AT2R and they concluded that that Ang-(1–9) is able to antagonize Ang II signalling in cardiomyocytes selectively via the AT2R, highlighting that Ang-(1–9), along with Ang-(1–7), makes up part of the counter-regulatory arm of the RAS. What remains to be determined is the downstream signalling effects from Ang-(1–9). Preliminary studies indicate that the classical pathways via PKC translocation and ERK1/2 activation [61–63] are not different between Ang II-, Ang-(1–7)- and Ang-(1–9) stimulated cells. Since the downstream signalling from the AT2R is unclear at present, future studies will be required to establish these mechanisms.

## 4. Role of the ACE2/Ang-(1–9) Axis in Hypertension

Crackower et al. [64] were the first to test ACE2 as the gene underlying the blood pressure locus on the X chromosome. They showed reduced expression of renal ACE2 in the salt-sensitive Sabra hypertensive rat compared with the normotensive rat. Both spontaneously hypertensive rats (SHR) and spontaneously hypertensive stroke-prone rats (SHRSP) rats showed reduced renal ACE2 protein levels compared with the normotensive Sabra and Wistar Kyoto (WKY) strains. Two other groups confirmed some of these findings showing lower renal ACE2 mRNA, protein, and activity in the SHR compared to WKY rats [65, 66]. However, other investigators were unable to detect any difference in renal ACE2 mRNA, protein, and activity between adult hypertensive rats and their normotensive controls [67]. 

Rentzsch et al. [68], assessed in SHRSP (that display reduced ACE2 mRNA and protein expression compared with control animals in the kidney) the role of ACE2 in the pathogenesis of hypertension. They generated transgenic rats on a SHRSP genetic background expressing the human ACE2 in vascular smooth muscle cells by the use of the SM22 promoter, called SHRSP-ACE2. In these transgenic rats, vascular smooth muscle cells (VSMC) expression of human ACE2 was confirmed by RNase protection, real-time RT-PCR, and ACE2 activity assays. Transgene ACE2 expression leads to significantly increased circulating levels of Ang-(1–7), a prominent product of ACE2. Mean arterial blood pressure was reduced in SHRSP-ACE2 compared to SHRSP rats, and the vasoconstrictive response to intraarterial administration of Ang II was attenuated. The latter effect was abolished by previous administration of an ACE2 inhibitor. To evaluate the endothelial function in vivo, endothelium-dependent and endothelium-independent agents such as acetylcholine and sodium nitroprusside, respectively, were applied to the descending thoracic aorta and blood pressure was monitored. Endothelial function turned out to be significantly improved in SHRSP-ACE2 rats compared to SHRSP. These data indicate that vascular ACE2 overexpression in SHRSP reduces hypertension probably by local Ang II degradation and by improving endothelial function [68]. 

A target gene therapy strategy holds significant potential to translate the available fundamental research of ACE2 into therapeutics. In fact, initial animal experiments have been extremely encouraging. For example, in SHR, viral-mediated ACE2 overexpression in the heart decreased high BP [69]. This strategy also preserved cardiac function, as well as left ventricular wall motion and contractility, and attenuated left ventricular wall thinning induced by myocardial infarction [70]. ACE2 overexpression in the rostral ventrolateral medulla causes significant decreases in BP and heart rate (HR) [71]. 

Compared with ACEIs and ARBs, the targeting of ACE2 has the following potential therapeutic advantages, first, it degradates both Ang I to generate Ang-(1–9) and Ang II to generate Ang-(1–7). Thus, targeting ACE2 would not only produce the antihypertrophic peptide Ang-(1–9) [52] and the vasoprotective/antiproliferative peptide Ang-(1–7) [72–74], but would also influence the vasoconstrictive/proliferative effects of the ACE/Ang II/AT1R axis [75]. Second, it is a multifunctional enzyme with many biologically active substrates [9, 20]. Third, unlike ARB/ACEI therapy, ACE2 is an endogenous regulator of the RAS [75]. Fourth, it is a part of the vasodilatory/antiproliferative axis of the RAS [20] and fifth, although treatment with ACEIs or ARBs indirectly increases ACE2 expression, direct activation of this enzyme could result in a better outcome in cardiovascular diseases [68, 75]. Thus, the activation of the ACE2 axis may be a novel therapeutic strategy in hypertension. 

So far, all attention has been focused on Ang-(1–7), that opposes the pressor, proliferative, profibrotic, and prothrombotic actions mediated by Ang II [76]. Experimental and clinical studies have demonstrated a role for the Ang-(1–7)/ACE2/Mas axis in the evolution of hypertension, the regulation of cardiovascular and renal function, and the progression of cardiovascular and renal disease including diabetic nephropathy [77]. Additional evidence suggests that a reduction in the expression and activity of this vasodepressor component may be a critical factor in mediating the progression of cardiovascular and renal disease. These findings support a role for the Ang-(1–7)/ACE2/Mas axis and, in particular, on its putative role as an ACE-Ang II-AT1 receptor counter-regulatory axis within the RAS [76, 77]. 

**Figure 1:**
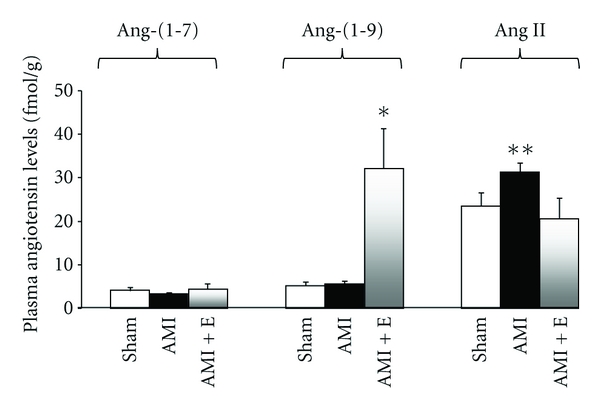
Plasma levels of Ang-(1–7), Ang-(1–9) and Ang II in rats with myocardial infarction treated with the ACE inhibitor enalapril (8 weeks). Increased plasma levels of Ang-(1–9) were observed in rats with myocardial infarction treated with the ACE inhibitor enalapril. Myocardial infarction was induced by coronary artery ligation. Data are presented as mean ± SEM (*n* = 12/group). AMI: acute myocardial infarction, E: enalapril. *P < 0.05 compared to both Sham and untreated myocardial infarction groups; **P < 0.05 compared to both Sham and enalapril-treated myocardial infarction groups. (adapted with permission from [51]).

Recently, the alternative angiotensin peptide, Ang-(1–9) has shown relevant biological functions. Ocaranza et al. [51] have observed increased ACE2 activity and Ang-(1–9) plasma levels in MI and sham rats treated with enalapril for 8 weeks while circulating Ang-(1–7) levels did not change in any phase after MI [51] (Figure 1). These findings support the hypothesis that, in this second arm of the RAS, ACE2 through Ang-(1–9) instead of Ang-(1–7), could act as a counterregulator of the first arm, where ACE catalyzes the formation of Ang II. 

**Figure 2:**
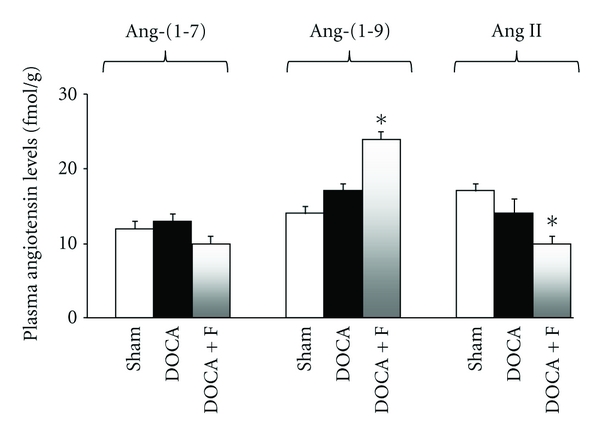
Plasma levels of Ang-(1–7), Ang-(1–9) and Ang II in DOCA salt hypertensive rats treated with the Rho kinase inhibitor fasudil. Increased plasma levels of Ang-(1–9) were observed in DOCA salt hypertensive rats treated with the Rho kinase inhibitor fasudil. Fasudil (100 mh/kg/day) by gavage was administered during 3 weeks, starting on the third week after DOCA administration. Data are presented as mean ± SEM (*n* = 8–11/group). DOCA: deoxycorticosterone, F: fasudil. *P < 0.05 compared to both Sham and untreated DOCA groups (adapted with permission from [78]).

 Besides, in experimental hypertension (DOCA salt model) and in normotensive sham animals, RhoA/Rho-kinase inhibition (a signaling pathway that participates in pathological cardiovascular and renal remodeling and also in blood pressure regulation) by fasudil reduced BP and increased vascular and plasma ACE2 enzymatic activity. At the same time, fasudil reduced Ang II and increased Ang-(1–9) plasma levels (Figure 2) [78]. No modifications were observed here in Ang-(1–7) levels despite increased ACE2 levels with RhoA/Rho-kinase inhibition [78]. Thus, RhoA/Rho-kinase inhibition, by increasing eNOS and/or by reducing both ACE and Ang II, does not activate the Ang-(1–7) pathway. This novel effect of RhoA/Rho-kinase inhibition on both ACE2 expression and Ang-(1–9) levels might additionally contribute to the antihypertensive effects of RhoA/Rho-kinase inhibitors. Besides, these results strongly suggest that in this experimental model, hypertension is more dependent on ACE2 and Ang-(1–9) levels than on ACE and Ang II levels. Therefore, this second RAAS axis through ACE2 and Ang-(1–9) could be an important target for the treatment of hypertension. 

## 5. Role of ACE2/Ang-(1–9) Axis in Vascular Remodeling

The vascular wall is continuously exposed to hemodynamic forces such as the luminal pressure and shear stress. Changes in these forces, either physiological or pathological, lead to functional and/or structural alterations of the vascular wall [79]. Acute changes in hemodynamic forces can modify vessel diameter. Chronic changes in hemodynamic forces result in structural alterations of the vessel wall, indicated by changes in wall diameter and thickness. In addition, changes in vascular structure are not solely determined by hemodynamic forces [80], but also by inflammatory responses and changes in extracellular matrix components [81]. Structural changes of the medial layer of the vascular wall during hypertension are termed “eutrophic remodeling” [82] and subsequently translate to other vascular pathologies. This involves an inward encroachment of the arterial wall thereby, reducing the diameter of the lumen [83]. 

Several RAAS components are involved in neointimal formation after vascular endothelial damage [84]. In particular, Rakugi et al. [85] observed that vascular endothelial damage results in the induction of vascular ACE. Their results suggested that inhibition of vascular ACE might be critical in the prevention of restenosis after balloon injury. Patients with previously untreated essential hypertension and eutrophic inward remodeling appears to respond to antihypertensive medication. Reduction in BP with drugs that block the RAAS such as ACEIs [86–88] or ARBs [86, 87, 89] and calcium channel antagonists [90] are able to reverse the eutrophic inward remodeling [88]. 

The protein and mRNA of ACE2 are expressed in human coronary arteries and arterioles and the vasa vasorum of most organs [9, 91]. Recently, ACE2 expression has also been observed in the large conduit arteries (aorta and carotid) in the HR [92]. ACE2 localizes preferentially in endothelial cells and arterial smooth muscle cells (SMCs) [9, 91]. As for the role of ACE2 in vascular remodeling, the effect of ACE2 on neointima formation has not yet been studied, but Ang-(1–7) infusion after balloon-catheter injury of the rat carotid artery reduced neointima formation [93]. This effect was probably mediated by its inhibition of vascular SMC proliferation [94]. In hypertensive animal models, ACE2 mRNA and protein were associated with immunoreactive Ang-(1–7) in the large conduit arteries of SHRs. Treatment with an ARB induced a fivefold increase in ACE2 mRNA and was associated with a significant increase in aortic Ang-(1–7) protein expression. This effect was associated with a decrease in aortic medial thickness, suggesting that this may be a protective mechanism in the prevention of cardiovascular events during hypertension [94]. Igase et al. [95] showed that ACE2 protein is expressed not only in the media of the carotid artery but also in the neointima of the balloon-injured carotid artery in SHR. The increase in ACE2 protein expression in the neointima following exposure of the rats to an ARB compared to vehicle was associated with a reduction in neointima thickness. These results lead to the hypothesis that there is a strong correlation between the increase in ACE2 protein in the injured carotid artery of SHR and vascular remodeling during blockade of Ang II receptors [95].

There is known the prothrombotic effect of Ang II [96, 97] and the antithrombotic action of Ang-(1–7) [98] in renovascular hypertensive rats. Thus, in this context, the question arises whether Ang-(1–9) effects are similar to Ang II or to Ang-(1–7) in in vivo conditions. Kramkowski et al. [99] described that Ang-(1–9) enhances electrically stimulated thrombosis in rats and that this effect was abolished by losartan—an antagonist of the AT1 receptor. The prothrombotic activity of Ang-(1–9) was accompanied by the enhancement of ex vivo platelet aggregation and in vitro Ang-(1–9) increased platelet aggregation. However, there are some points in this paper that should be clarified. First, thrombus formation was initiated by electrical stimulation producing arterial injury that is unrelated to a clinical situation. Second, the prothrombotic effect of Ang-(1–9) was much weaker, to the prothrombotic action of Ang II [96, 97]. Third, Ang-(1–9) slightly increased platelet aggregation in in vitro conditions.

On the contrary Ocaranza et al. [78] showed that by inhibiting the RhoA/Rho-kinase pathway with fasudil, gene expression and enzymatic ACE activity and plasma levels of Ang II were reduced (Figure 2) and whereas aortic gene expression and ACE2 activity were importantly increased. Simultaneously, plasma levels of Ang-(1–9) (Figure 2), mRNA eNOS levels increased and the aortic overexpression of the remodeling promotion proteins TGF-*β*1, PAI-1, and MCP-1 as well as the increased aortic NADPH oxidase activity and O^2−^ production were reduced, as a consequence of direct RhoA/Rho-kinase inhibition [100]. This novel effect of RhoA/Rho-kinase inhibition on ACE2 gene expression, enzymatic activity, and Ang-(1–9) levels might additionally contribute to its benefits in hypertension, atherosclerosis, and in cardiovascular and renal pathologic remodeling. This is the first observation concerning a pharmacologic ACE2 and Ang-(1–9) levels activator, both in normotensive and in hypertensive animals, one of the most interesting findings of that study (Figure 2). Additionally, in experimental hypertension, direct RhoA/Rho-kinase inhibition also normalizes overexpression of genes that promote vascular remodeling. Interestingly, the observed changes in ACE/ACE2 and in Ang-(1–9) levels were present only during fasudil treatment both in sham and in the DOCA hypertensive rats [78]. Thus, vascular remodeling could be more dependent on the tissue ACE2/Ang-(1–9) axis than on Ang-(1–7) levels in normotensive as well as in hypertensive rats.

In vessels, new members of the RAS have been detected, including ACE2, Ang-(1–7) and Mas. Vascular ACE2 is functionally active and generates Ang-(1–7) from Ang II. Ang-(1–7) is found in the endothelium and vascular wall [101–103] and immunohistochemical staining shows abundant presence in aortic perivascular adventitial tissue [104, 105]. Ang-(1–7), by binding to receptor Mas on endothelial cells, opposes Ang II actions by mediating vasodilation, growth-inhibition, antiinflammatory responses, antiarrhythmogenic and antithrombotic effects [33, 68] through NOS-derived NO production, activation of protein tyrosine phosphatases, reduced MAPK activation and inhibition of NADPH oxidase-derived generation of reactive oxygen species (ROS) [106, 107]. Overexpression of ACE2 in the vascular wall of SHR is associated with improved endothelial function and attenuated development of hypertension [68]. Ang-(1–7)-Mas can hetero-oligomerize with AT1R, thereby inhibiting Ang II actions. The ACE2/Ang-(1–7)-Mas axis is now considered as a counter-regulatory system to the ACE-Ang II-AT1R axis in the vasculature [107], although some evidence indicates that Ang-(1–7) may also promote fibrosis and inflammation in certain conditions [108, 109].

## 6. Role of the ACE2/Ang-(1–9) Axis in Cardiac Remodeling

After myocardial injury or in response to chronically increased hemodynamic load, cardiac mass increases as a result of cardiomyocyte hypertrophy and ventricular wall thickening. Initially these changes are compensatory mechanisms which help to maintain ejection performance and heart function. With continued hemodynamic overload the heart becomes dilated and its walls thinner, resulting in a geometry that contributes to systolic dysfunction by increasing wall stress [110]. At the cellular level, cardiac myocytes increase in size (hypertrophy), rearrange within the myocardial matrix (cell slippage), and die, to be replaced by fibrous tissue, which include fibroblasts and collagen. These changes are collectively referred to as “remodeling” [111]. Cardiac remodeling has been consistently associated with an impaired prognosis in patients with hypertension, MI and chronic heart failure (CHF) [112]. 

Despite recent advances in our understanding of the ACE2/Ang-(1–7)/axis, the functional role of ACE2 in the heart is somewhat controversial. Crackower et al. [64] originally reported a progressive reduction in LV contractile function in ACE2-null mice without significant changes in fibrosis, left ventricular and cardiac myocyte hypertrophy, or in mean arterial pressure [64]. Interestingly, whereas plasma and tissue levels of Ang II were increased, a decrease in blood pressure was only observed in 6-month-old male ACE−/− homozygote mice but not in age-matched females or 3-month-old males. Conversely, Gurley et al. [113] reported that ACE2 deletion enhanced the susceptibility to Ang II-induced hypertension but had no effect on cardiac structure or function [113]. Huentelman et al. [114] showed that the ACE2 overexpression protects the heart from Ang II-induced hypertrophy and fibrosis. More recently, in SHR hypertensive rats Díez-Freire et al. by using lentiviral-based ACE2 gene transfer, attenuated cardiac fibrosis and hypertrophy [70] and also improved LV and remodeling after experimental MI [115]. Finally, Yamamoto et al. [116] reported that ACE2 deletion exacerbated pressure overload-induced cardiac dysfunction and remodeling that was associated with increased intracardiac Ang II levels and AT1R activation. The reasons for these discrepancies seem to be: (a) the genetic background of the mice used for ACE2 gene deletion [113], (b) global versus tissue-specific ACE2 manipulation, or (c) the cardiac responses were monitored under basal or pathophysiological conditions.

In MI Ocaranza et al. [51] observed that (a) circulating and LV enzymatic activities of ACE2 were downregulated in the long-term phase of LV dysfunction in rats, (b) these effects were prevented by the conventional ACE inhibitor enalapril, (c) plasma Ang-(1–9) levels were significantly increased when MI rats or sham-operated rats were treated with enalapril for 8 weeks but circulating Ang-(1–7) levels did not change at that time (Figure 1) [51]. Based on these findings, it was proposed in this model of HF, that Ang-(1–9) rather than Ang-(1–7) acts as a counterregulator of Ang II [51].

Recently, in MI rats randomized to receive either vehicle, the ACEI enalapril, or the ARB candesartan for 8 weeks, Ocaranza et al. [52] observed that both drugs prevented LVH and increased plasma Ang-(1–9) levels by several folds. Ang-(1–9) levels correlated negatively with different LVH markers with or without adjustment for BP reduction. This effect was specific as neither Ang-(1–7), Ang II nor bradykinins were correlated with LVH. Chronic administration of Ang-(1–9) to MI rats by osmotic minipumps versus vehicle for two weeks decreased plasma Ang II levels, inhibited ACE activity and also prevented cardiac myocyte hypertrophy. Because there are in vitro evidences that the incubation of Ang-(1–9) with ACE generates Ang-(1–7) [9], and Ang-(1–7) negatively regulates hypertrophy [34, 117], the authors used the Ang-(1–7) receptor blocker A779 to investigate whether Ang-(1–7) could mediate the effects of Ang-(1–9). Even though A779 was bioactive, with significant increase in circulating Ang-(1–7) levels by 2.7 fold, this compound did not modify the Ang-(1–9)-dependent suppression of cardiac myocytes hypertrophy induced by MI [52]. In in vitro experiments with cardiac myocytes incubated with norepinephrine (10 *μ*M) or with IGF-1 (10 nM), Ang-(1–9) also prevented hypertrophy and this effect was not modified by the coincubation with Ang-(1–9) and A779 [52]. 

 To further understand the role of Ang-(1–9) compared to Ang-(1–7) in cardiomyocyte hypertrophy, Flores-Muñoz et al. [56] studied Ang-(1–9) effects in rat neonatal H9c2 and in rabbit left ventricular cardiomyocytes. Cardiomyocyte hypertrophy was stimulated with Ang II or vasopressin, significantly increasing cell size by approximately 1.2-fold as well as stimulating expression of the hypertrophy gene markers atrial natriuretic peptide, brain natriuretic peptide, *β*-myosin heavy chain and myosin light chain (2- to 5-fold). Both Ang-(1–9) and Ang-(1–7) were able to block hypertrophy induced by either agonist. The effects of Ang-1–9) were not inhibited by captopril, supporting previous evidence that Ang-(1–9) acts independently of Ang-(1–7). The authors investigated receptor signalling via angiotensin type 1 and type 2 receptors (AT1R, AT2R) and Mas. The AT1R antagonist losartan blocked Ang II-induced, but not vasopressin-induced, hypertrophy. Losartan did not block the antihypertrophic effects of Ang-(1–9), or Ang-(1–7) on vasopressin-stimulated cardiomyocytes. The Mas antagonist A779 efficiently blocked the antihypertrophic effects of Ang-(1–7), without affecting Ang-(1–9). Furthermore, Ang-(1–7) activity was also inhibited in the presence of the bradykinin type 2 receptor antagonist HOE140, without affecting Ang-(1–9). Moreover, Flores-Muñoz et al. [56] observed that the AT2R antagonist PD123,319 abolished the antihypertrophic effects of Ang-(1–9), without affecting Ang-(1–7), suggesting Ang-(1–9) signals via the AT2R. Radioligand binding assays demonstrated that Ang-(1–9) was able to bind the AT2R (pKi = 6.28 ± 0.1). The data indicate that ACE2/Ang-(1–9) axis, acting as a counterregulator of Ang II, is an effective, and possibly direct novel anticardiac hypertrophy axis. 

## 7. Conclusions

**Figure 3:**
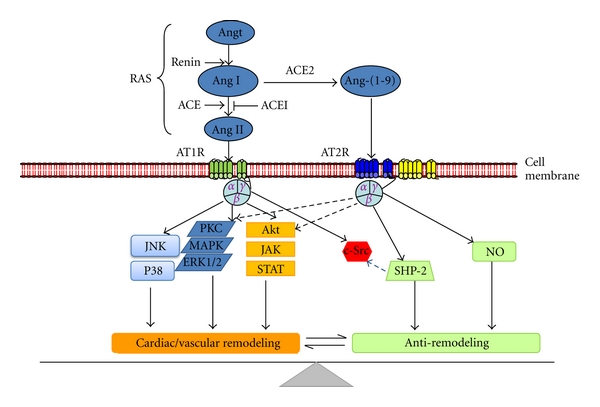
Signaling events and cellular effects induced by Ang II via AT1R and opposing effects of Ang-(1–9) acting through AT2R. Proposed Ang-(1–9)-dependent mechanisms that antagonize the cardiovascular remodeling effects of Ang II. ACE2 can directly cleave Ang I to form Ang-(1–9). This peptide activates the AT2R to initiate signaling pathways that antagonize AT1R-mediated tyrosine kinase cascades. In this simplified scenario, Ang-(1–9) increases SHP-1 tyrosine phosphatase activity to inactivate src-dependent signaling. AT2R activation also acts other pathways such as NO-AKT. AT1R: Ang II type 1 receptor; AT2R: Ang II type 2 receptor; ERK1/2: extracellular signal-regulated kinase 1/2; JAK: Janus-activated kinase; MAPK: mitogen-activated protein kinase; p38: p38 MAPK; PKC: protein kinase C; STAT: signal transducer and activator of transcription; NO: nitric oxide; SHP-1: protein tyrosine phosphatase SH2 domain-containing phosphatase 1; MEK: mitogen/ERK kinase. Solid arrows indicates activation broken arrows indicates inactivation.

Pharmacological treatments based on the RAS blockade are used extensively for the treatment of hypertension and CV remodeling. However, in spite of their success in pharmacological blockade of the RAS, the prevalence of end-organ damage has risen steadily in the last several decades. These observations indicate that novel and innovative approaches must be used in an attempt to promote a more effective treatment for the residual CV remodeling. In this environment, the ACE2/Ang-(1–9) axis is an important target, that is critical in tipping the balance of vasoconstrictive/proliferative to vasodilatory/antiproliferative axis of the RAS. Conceptually, the ACE2/Ang-(1–9)/AT2 axis balances the adverse effects of the ACE-Ang II-AT1 receptor axis (Figure 3). Accumulating evidence suggests that ACE2 expression and Ang-(1–9) levels are altered in diastolic and systolic dysfunction and remodeling and the activation of the ACE2/Ang-(1–9) axis protects the heart and vessels from cardiovascular remodeling. In conclusion, the noncanonical RAS arm has new biological effector Ang-(1–9) to counterregulate the classical RAS.
